# Snack food intake in ad libitum fed rats is triggered by the combination of fat and carbohydrates

**DOI:** 10.3389/fpsyg.2014.00250

**Published:** 2014-03-31

**Authors:** Tobias Hoch, Monika Pischetsrieder, Andreas Hess

**Affiliations:** ^1^Food Chemistry Unit, Department of Chemistry and Pharmacy, Emil Fischer Center, Friedrich-Alexander Universität Erlangen-NürnbergErlangen, Germany; ^2^Institute of Experimental and Clinical Pharmacology and Toxicology, Emil Fischer Center, Friedrich-Alexander Universität Erlangen-NürnbergErlangen, Germany

**Keywords:** snack food, food intake, macronutrients, eating behavior, rat, preference test

## Abstract

Snack food like potato chips substantially contributes to energy intake in humans. In contrast to basic food, snacks are consumed additionally to other meals and may thereby lead to non-homeostatic energy intake. Snack food is also frequently associated with hedonic hyperphagia, a food intake independent from hunger. Analysis of brain activity patterns by manganese-enhanced MRI has previously revealed that the intake of potato chips in ad libitum fed rats strongly activates the reward system of the rat brain, which may lead to hedonic hyperphagia. The purpose of the present study was to develop a two-choice preference test to identify molecular determinants of snack food triggering extra food intake in ad libitum fed rats. Different kinds of test food were presented three times a day for 10 min each time. To minimize the influence of organoleptic properties, each test food was applied in a homogenous mixture with standard chow. Food intake as well as food intake-related locomotor activity were analyzed to evaluate the effects induced by the test foods in the two-choice preference test. In summary, fat (F), carbohydrates (CH), and a mixture of fat and carbohydrates (FCH) led to a higher food intake compared to standard chow. Notably, potato chip test food (PC) was highly significantly preferred over standard chow (STD) and also over their single main macronutrients F and CH. Only FCH induced an intake comparable to PC. Despite its low energy density, fat-free potato chip test food (ffPC) was also significantly preferred over STD and CH, but not over F, FCH, and PC. Thus, it can be concluded that the combination of fat and carbohydrates is a major molecular determinant of potato chips triggering hedonic hyperphagia. The applied two-choice preference test will facilitate future studies on stimulating and suppressive effects of other food components on non-homeostatic food intake.

## INTRODUCTION

Savory snacks like potato chips counted among the seven major contributors to energy intake in children and adolescents in the US during the last 21 years (Slining et al., 2013). Snack food is not part of our basic diet, but is frequently consumed additionally to other meals. Moreover, snacks show only a weak satiety effect and their calorie content is not or only partially compensated by reduced ingestion of standard meals (Whybrow et al., 2007; Chapelot, 2011). Thus, it can be concluded that snack food consumption leads to increased total energy intake. The so-called hedonic food intake is independent from hunger, may overrule the homeostatic energy balance and therefore lead to hyperphagia, i.e., food intake beyond satiety (Berthoud, 2011).

Several studies suggest that certain types of food can induce similar non-homeostatic energy intake in rats as in humans indicating the existence of a highly phylogenetically conserved neural regulation mechanism of food intake. For example, it has been shown that rats that have access to a cafeteria diet take up twice as much energy as rats with access to standard chow only. Additionally, the feeding pattern changed from meal-based food intake to snacking-based food intake (Martire et al., 2013). In a similar way, ad libitum fed rats with additional access to potato chips showed higher energy intake than rats with additional access to standard chow only (Hoch et al., 2013).

Several studies investigated the underlying physiological mechanisms which are related to non-homeostatic intake of palatable food. Recently, it was shown that a cafeteria diet affects the reward system in the rat brain (Epstein and Shaham, 2010) and that the snack food potato chips modulates the activity of brain areas that respond to cues mainly regulating reward and addiction, food intake, locomotor activity, and sleep (Hoch et al., 2013). On a molecular level, various systems are involved in the regulatory mechanisms of non-homeostatic food intake including hormones, dopamine, melanocortins or other signal molecules (Berthoud, 2011; Pandit et al., 2011; Alsio et al., 2012). For example, the hedonic intake of several snack foods seems to be regulated by the endogenous opioid system, because the opioid antagonist naltrexone attenuated the conditioned place preference induced by different solid snack foods in ad libitum fed rats (Jarosz et al., 2006). The endocannabinoid system of the gut may be an important regulator of fat intake (DiPatrizio et al., 2011).

Nevertheless, the molecular food determinants that trigger non-homeostatic food intake are not fully characterized. Several studies used a cafeteria diet as palatable feed, which contains a selection of different articles such as cakes, pasta, potato chips, cookies, cheese, or nuts (Prats et al., 1989; Martire et al., 2013). In other studies, single food items were used, such as potato chips (Hoch et al., 2013) or Froot Loops^®^ cereals (Jarosz et al., 2006). Excessive food intake was mostly related to the energy-, fat-, or sugar content of the food. Additionally, sensory properties were also suggested to have an influence: in well-fed rats, food intake was rather induced by the food’s palatability or sensory properties, whereas the calorie content seemed to be the main contributor in rats with negative energy balance (Scheggi et al., 2013).

The aim of the current study was, therefore, to apply a two-choice food preference test that can be used to determine the activity of single components of snack food to induce food intake. Two-choice preference tests have been previously applied, for example, to test the preference of rats for food flavors, the influence of galanin administration on food choice or the relative palatability of sucrose/oil emulsions (Naim et al., 1986; Smith et al., 1996). For our purpose, a two-choice preference protocol for solid foods was modified in a way that parts of a reference powdered standard chow (STD) were replaced either by the snack food or by single components in the concentration present in the snack food. Thus, the different test foods could be tested against the STD reference and against each other. As a model for a snacking situation, the test foods were presented each time for 10 min only and the rats always had ad libitum access to standard chow pellets. This test system was then applied to analyze the effects of the macronutrients on the intake of potato chips.

## MATERIALS AND METHODS

### ETHIC STATEMENT

This study was carried out in strict accordance with the recommendations of the Guide for the Care and Use of Laboratory Animals of the National Institutes of Health. The protocol was approved by the Committee on the Ethics of Animal Experiments of the Friedrich-Alexander-Universität Erlangen-Nürnberg (FAU).

### ANIMALS

Behavioral tests were conducted with 18 rats in total. Initially, the tests were conducted with eight male Wistar rats (two cages with four animals each, initial weight 210 ± 8 g, kept in a 12/12 h dark/light cycle, purchased from Charles River, Sulzfeld, Germany). The majority of experiments were reproduced with 10 male Sprague Dawley rats (two cages with five animals each, initial weight 181 ± 14 g, kept in 12/12 h dark/light cycle, purchased from Charles River, Sulzfeld, Germany). The rats had access to STD pellets (Altromin 1324, Lage, Germany) and tap water ad libitum throughout the whole study.

### TEST FOODS

**FIGURE 1:**
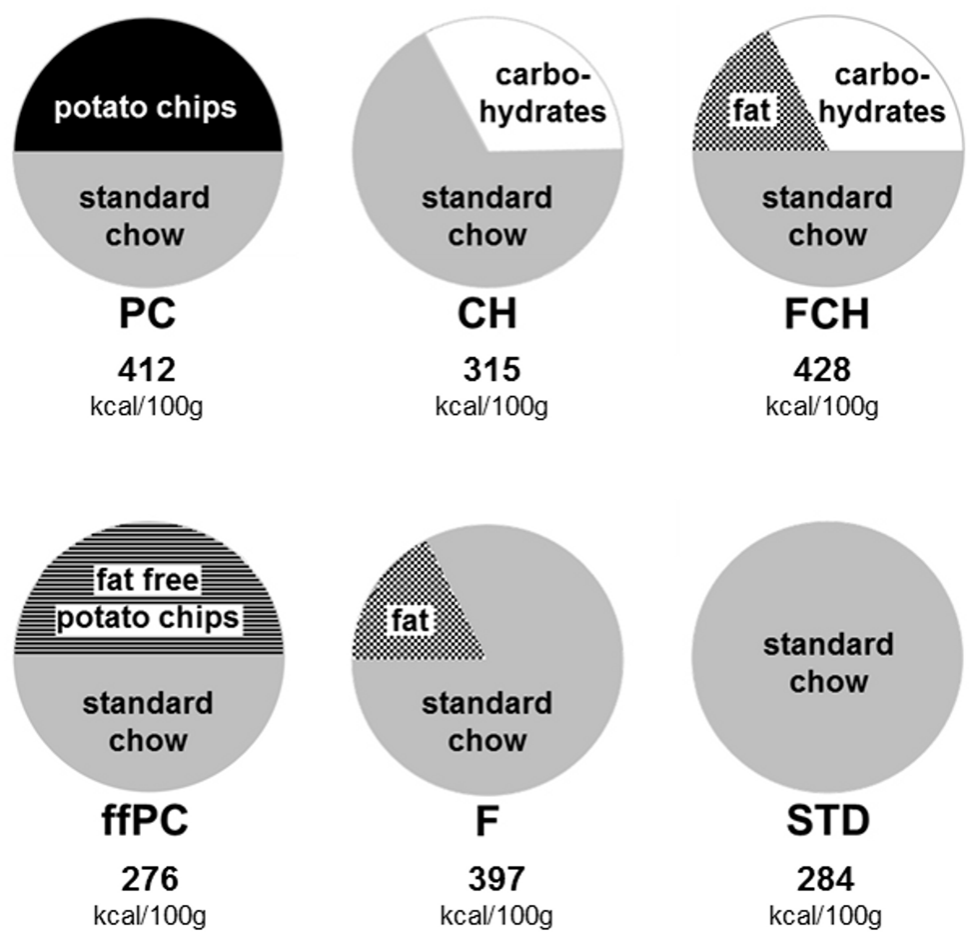
**Composition (percent by weight) and energy content (kcal/100 g) of the test foods: potato chips (PC), fat-free potato chips (ffPC), carbohydrate content of PC (CH), fat content of PC (F), fat and carbohydrate mixture (FCH), and powdered standard chow (STD)**.

All test foods were prepared, mixed, and crushed in a food processor to ensure homogeneity and a similar texture. The test food PC consisted of powdered STD (Altromin 1321, Lage, Germany) in a mixture with 50% potato chips (“PFIFF Chips Salz”, unflavored, salted, without added taste compounds or taste enhancers, purchased from a local supermarket; 49% carbohydrates, 35% fat, 6% protein, 4% dietary fiber, 1.8% salt). The test food ffPC contained 50% fat-free potato chips (“Lay’s Light Original^®^”, with the fat substituent olestra (OLEAN^®^), unflavored, salted, without added taste compounds or taste enhancers, purchased in a supermarket in the USA; 61% carbohydrates, 7% protein, 3.4% dietary fiber, 1.7% salt, 0% fat) in powdered STD. In order to test the combined influence of the macronutrients fat and carbohydrate on the palatability of potato chips, a model of the potato chips (FCH) was prepared, which consisted of 50% powdered STD and the fat and carbohydrate components of potato chips. The remaining part of the potato chips (proteins, fiber, salt, and unidentified components) was replaced by carbohydrates instead of STD in order to match the energy density of the model and PC as closely as possible. Thus, FCH consisted of 50% STD, 17.5% fat (sunflower oil, purchased from a local supermarket) and 32.5% carbohydrates (dextrin from maize starch, maltodextrine, Fluka, Taufkirchen, Germany). Additionally, the fat and carbohydrate portions of the test food FCH were tested separately. Thus, for testing the influence of the fat content (F), 17.5% fat was mixed with 82.5% STD. The effect of the carbohydrate content (CH) was tested with food consisting of 32.5% carbohydrates and 67.5% STD. The energy density of the different test foods was calculated based on the manufacturer’s labeling. The calculated values and the composition of the test foods are illustrated in **Figure 1**.

### EXPERIMENTAL DESIGN

**FIGURE 2:**
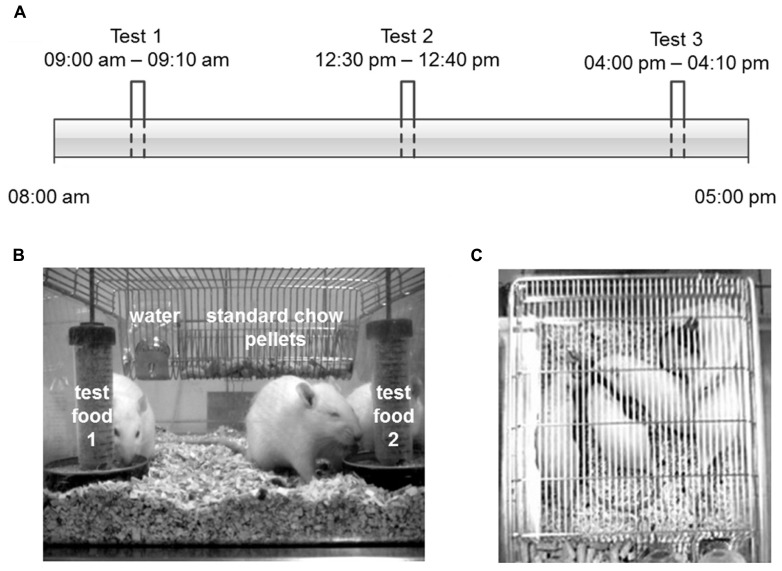
**Overview on the study design: (A) Schedule for the three separate two-choice preference tests on one day at 9 am, 12.30 pm and 4 pm. (B)** Front view of the cage during the two-choice preference tests with the two additional test food dispensers (test food 1 and 2), which were presented three times a day. In the background the STD pellets as well as tap water are visible, which were constantly available ad libitum. **(C)** View from above on the cage during a preference test for the evaluation of the feeding-related locomotor activity.

For the two-choice preference tests, test foods were presented three times per day (at 9 am, 12:30 pm, and 4 pm), each time for 10 min (**Figure 2A**) in two additional food dispensers (**Figure 2B**). The test food intake was determined by the weight difference of the food dispensers before and after each access period. Energy intake was calculated by multiplying these amounts of ingested food with the respective energy contents. The relative food and energy intake were calculated by dividing the ingested amount of food or energy of the particular test food by the sum of the two test foods provided. The position of the food dispensers and the food filled into a particular dispenser were changed for every test to avoid the influence of place preferences. Additionally, the feeding-related locomotor activity of the rats was measured. For that purpose, pictures were taken every 10 s via webcams placed above the cages (**Figure 2C**). The resulting 60 pictures recorded per single period of food access were evaluated by counts: one count was defined as “one rat takes food from one food dispenser”. The ingested amounts of food, energy as well as the counts were used to calculate the relative contribution of each test food to the total food intake additionally to the standard chow pellets in every single test. Each experiment was performed simultaneously in two cages on two consecutive days with three tests per day. Selected food combinations were repeated on up to six days. The following experiments were performed with two different animal cohorts: PC vs. CH, PC vs. F, PC vs. FCH, F vs. CH, FCH vs. CH, FCH vs. F, ffPC vs. PC, ffPC vs. CH, ffPC vs. F, and ffPC vs. FCH.

### STATISTICAL ANALYSIS

For statistical analysis, we calculated the percentage of the test foods, which were ingested in one cage during every single 10 min preference test, related to the total intake from both test food containers. The preference tests were performed as 6–50 single tests (10 min each) with 2–4 independent animal groups (cages) comprising 4–5 individuals each. A one-way repeated measures analysis of variance (ANOVA) with the variable “test days” did not reveal any significant influence of this variable (*p* < 0.05) for the majority of the test conditions (see Results and Discussion for exceptions). For the tested combinations of PC vs. FCH (*p* = 1.06 × 10^-^^7^) and PC vs. F (*p* = 4.13 × 10^-^^5^) ANOVA showed a significant influence of the variable “test days”. Consequently, we analyzed these data separately for each day.

**FIGURE 3:**
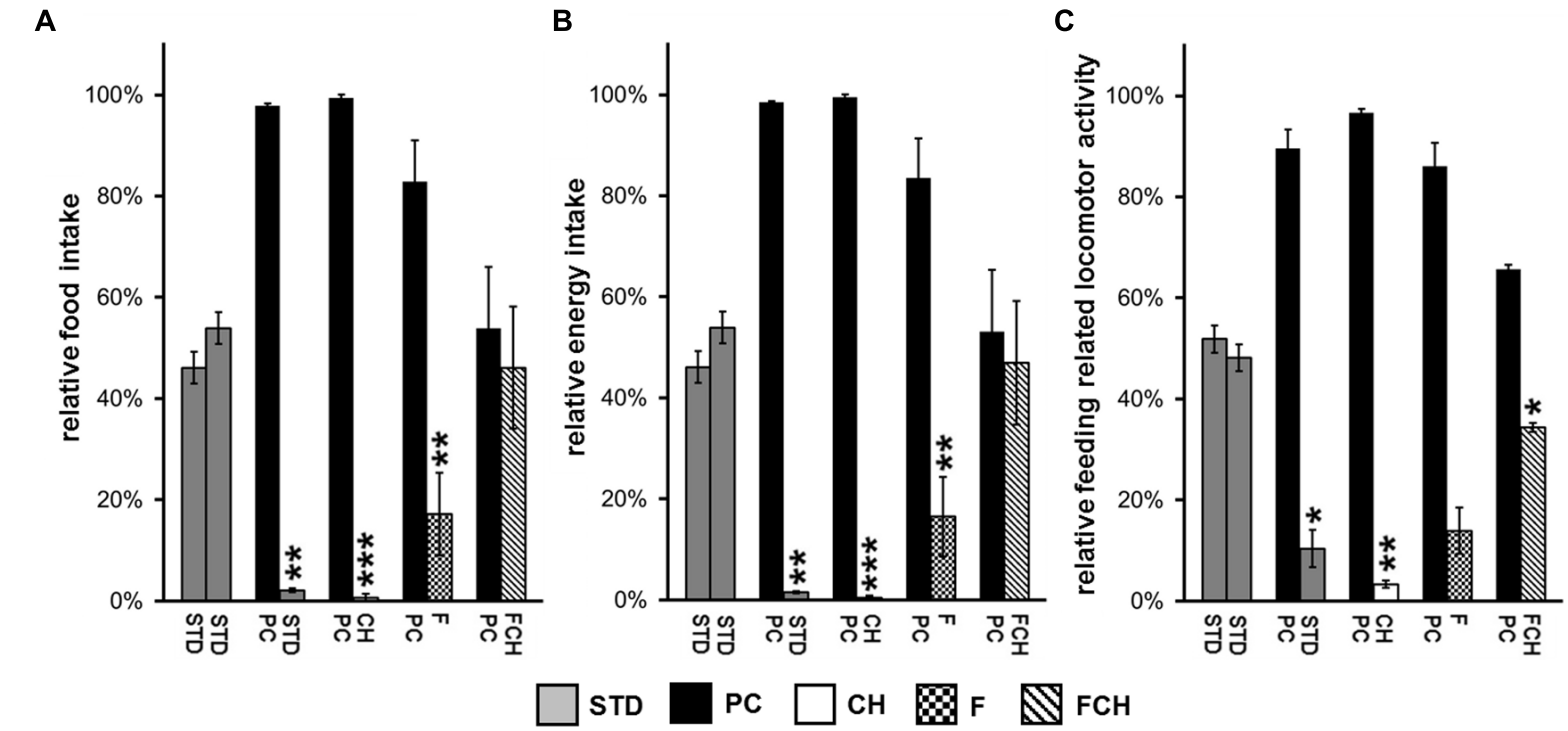
**Two-choice preference tests between the different test foods: (A) Relative food intake, **(B)** relative energy intake, and **(C)** relative feeding-related locomotor activity testing standard chow (STD) in both food containers or potato chips (PC) vs. STD as well as their major macronutrients carbohydrates (CH), fat (F), and fat and carbohydrates (FCH).** Mean ± standard deviation of the relative food/energy intake or locomotor activity of the independent animal groups (cages) are shown. ***p < 0.001, **p < 0.01, *p < 0.05.

**FIGURE 4:**
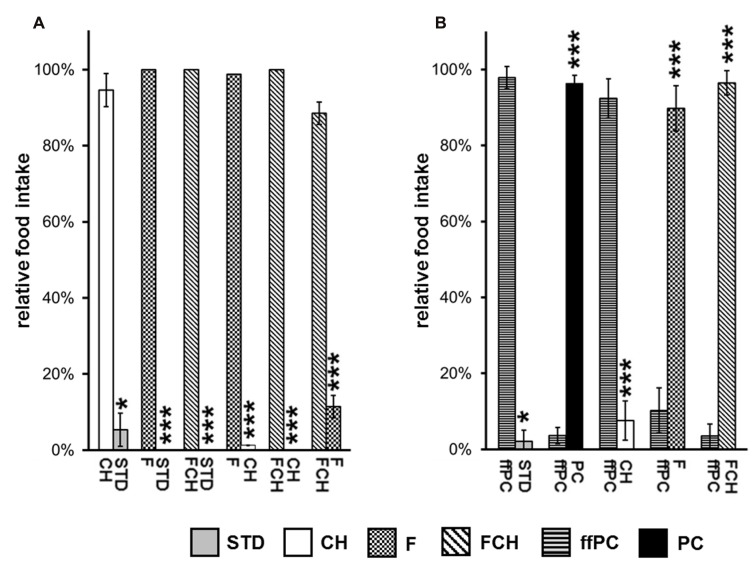
**Relative food intake during two-choice preference tests (A) applying the major macronutrients of potato chips (PC), carbohydrates (CH), fat (F) as well as fat and carbohydrates (FCH), and standard chow (STD). (B)** Two-choice preference test of fat-free potato chips (ffPC) vs. STD, PC, and the macronutrients (CH, F, FCH) of PC. Mean ± standard deviation of the relative food intake of the independent animal groups (cages) is shown. ***p < 0.001, *p < 0.05.

**FIGURE 5:**
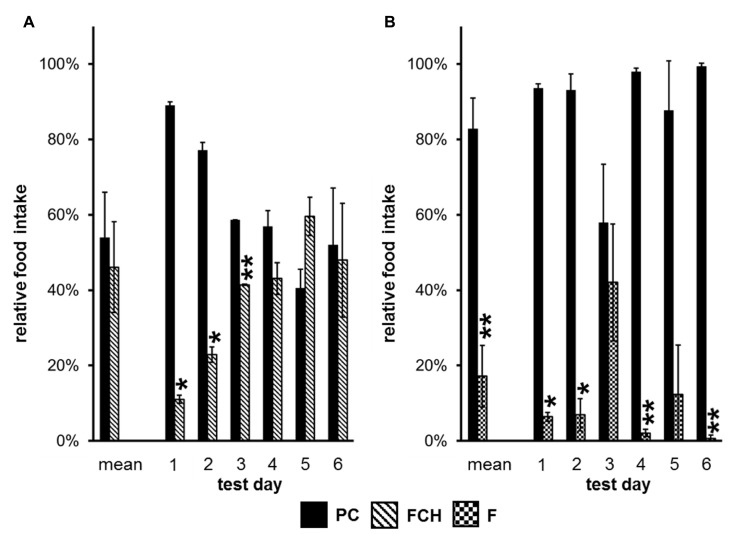
**(A) Relative food intake (mean and single values of six different test days) during two-choice preference tests of potato chips (PC) vs. the mixture of fat and carbohydrates (FCH), and **(B)** PC vs. the fat content of potato chips (F).** Mean ± standard deviation of the relative food intake of the independent animal groups (cages) is shown. **p < 0.01, *p < 0.05.

Significances of food intake for a given test food combination were calculated by a paired, two-sided Student’s *t*-test using Analysis ToolPak, Microsoft Excel 2013. The mean values of the single tests were calculated for the independent groups (cages) and used for statistical testing (*n* = 2–4). The data are presented in **Figures 3–5** and in **Tables 1–4**. A *p*-value < 0.05 was considered to be significant.

**Table 1:** Statistical data for “food intake” **(A)** “energy intake” **(B)** and “locomotor activity” **(C)** of preference tests with two of the following test foods: powdered standard chow (STD), potato chips (PC), carbohydrates (CH), fat (F), and the mixture of fat and carbohydrates (FCH).

Food	Mean (%)	SD (%)	df	*p*-Value	*t*-Value	Number of single tests	Number of animals	See figure
**(A) Food intake**
STD	46	3	1	0.3311	–1.746	12	8	3A
STD	54	3
PC	98	0	1	0.0035	181.0	12	10	3A
STD	2	0
PC	99	1	3	8.8 × 10^-7^	135.7	36	18	3A
CH	1	1
PC	83	8	3	0.0040	8.050	48	18	3A
F	17	8
PC	54	12	3	0.5634	0.647	50	18	3A
FCH	46	12
**(B) Energy intake**
STD	46	3	1	0.3311	–1.746	12	8	3B
STD	54	3
PC	98	0	1	0.0022	291.0	12	10	3B
STD	2	0
PC	100	1	3	3.4 × 10^-7^	186.9	36	18	3B
CH	0	1
PC	84	8	3	0.0034	8.555	48	18	3B
F	16	8
PC	53	12	3	0.6458	0.509	50	18	3B
FCH	47	12
**(C) Locomotor activity**
STD	52	3	1	0.5089	0.973	6	8	3C
STD	48	3
PC	90	4	1	0.0421	15.08	12	10	3C
STD	10	4
PC	97	1	1	0.0070	90.58	24	10	3C
CH	3	1
PC	86	5	1	0.0568	11.19	36	10	3C
F	14	5
PC	66	1	1	0.0262	24.31	36	10	3C
FCH	34	1

*Mean and SD of the relative food/energy intake or locomotor activity of the independent animal groups (cages) are presented. The *p*- and *t*-values are calculated via two-sided paired Student’s *t*-test with df as the degree of freedom. Furthermore, number of single tests and number of animals are given.*

Statistical analysis regarding the energy intake and feeding-related locomotor activity was performed accordingly. The overall correlation between food intake and feeding-related locomotor activity was determined by a linear regression analysis between food intake [g] and the feeding-related locomotor activity [counts] of every single test over all tested conditions.

## RESULTS

It is well-established that snack food like potato chips is able to trigger non-homeostatic food intake. The purpose of the present study was to develop a test system for the identification of the particular snack food components which are responsible for these processes. The developed test system was then applied to investigate the contribution of the main macronutrients (carbohydrates and fats) to the intake of snack food.

To develop a screening assay, the potential of a test food to induce food intake in non-deprived ad libitum fed rats was used as readout. The feeding activity was recorded by two independent parameters. First, the amount of ingested food was weighed. Additionally, feeding-related locomotor activity was recorded by a camera. Both methods showed a very high correlation between all tested conditions (*r* = 0.9204, *R*^2^ = 0.8471, *p* < 0.001). Feeding activity displayed as relative food intake or as relative energy intake provided similar results, which only differed by ≤3 percentage points as exemplified in **Figures 3A,B**.

Since the absolute amount of test food intake varied from day to day and was, for example, dependent on the age of the animals (data not shown), a two-choice preference test was applied (**Figure 2B**), which recorded the food intake in relation to a reference food. Although the feeding experiments were performed during the light cycle of the day, i.e., the resting phase of the rats (Hoch et al., 2013), considerable additional food intake was observed, which was dependent on the composition of the test food. A lack of side- or place-preference was observed when powdered STD was provided in both of the food dispensers resulting in a similar food and energy intake from both dispensers without significant difference (*p* = 0.3311, **Figures 3A,B; Tables 1A,B**). Additionally, a similar feeding-related locomotor activity on both food dispensers was observed (*p* = 0.5089, **Figure 3C**; **Table 1C**). No significant variance (*p* < 0.05) of the relative preferences for one of the two presented test foods between the test days could be observed for any of the test conditions, except for PC vs. FC and PC vs. F. These exceptions are described below in more detail.

The first experiment, when PC was tested against STD, resulted in an almost exclusive ingestion of PC (**Figures 3A,B; Tables 1A,B**). Next, the contribution of the two major macronutrients of PC, namely carbohydrate and fat, on the food intake was studied. For this purpose, the carbohydrate (test food CH) or fat (test food F) content as described above was added to STD. Both test foods CH and F induced a significantly (CH: *p* < 0.05, F: *p* < 0.001, **Figure 4A**; **Table 2**) higher intake than STD, whereby F prevailed against CH (*p* < 0.001, **Figure 4A**; **Table 2**), but neither CH nor F were able to induce food intake similar to PC (**Figures 3A,B**; **Tables 1A,B**). The results indicate that the activity of potato chips to induce food intake in non-deprived rats cannot be explained by the fat content or the carbohydrate content of potato chips alone.

**Table 2:** Statistical data for “food intake” of preference tests with two of the following test foods: carbohydrates (CH), powdered standard chow (STD), fat (F), the mixture of fat and carbohydrates (FCH), fat-free potato chips (ffPC), and potato chips (PC).

Food	Mean (%)	SD (%)	df	*p*-Value	*t*-Value	Number of single tests	Number of animals	See figure
CH	95	4	1	0.0439	14.46	12	10	4A
STD	5	4
F	100	0	1	2.7 × 10^-5^	23997	12	10	4A
STD	0	0
FCH	100	0	1	5.3 × 10^-5^	11997	12	10	4A
STD	0	0
F	99	0	3	1.8 × 10^-7^	229.6	24	18	4A
CH	1	0
FCH	100	0	1	5.3 × 10^-5^	11997	12	10	4A
CH	0	0
FCH	89	3	3	0.0001	26.27	24	18	4A
F	11	3
ffPC	98	3	1	0.0278	22.86	12	10	4B
STD	2	3
ffPC	4	2	3	2.7 × 10^-5^	–43.25	24	18	4B
PC	96	2
ffPC	92	5	3	0.0005	16.33	24	18	4B
CH	8	5
ffPC	10	6	3	0.0009	–13.36	24	18	4B
F	90	6
ffPC	4	3	3	8.8 × 10^-5^	–29.21	24	18	4B
FCH	96	3

* Mean and SD of the relative food intake of the independent animal groups (cages) are presented. The *p*-and *t*-values are calculated via two-sided paired Student *t*-test with df as the degree of freedom. Furthermore, number of single tests and number of animals are given.*

However, when the combined fat- and carbohydrate-fractions of potato chips were added to standard chow, the intake of this test food FCH was similar (**Figures 3A,B**; **Tables 1A,B**) and the feeding-related locomotor activity only slightly lower compared to PC (**Figure 3C**; **Table 1C**). Similar to PC, FCH was also almost exclusively ingested when presented in a preference test against F or CH (**Figure 4A; Table 2**).

Thus far, the present results indicate that the effect of potato chips to increase food intake in non-deprived rats is caused by its calorie content, which is essentially mediated by the fat and carbohydrate content. For a further test of this hypothesis, the feeding activity of ffPC was compared to the other test foods (STD, PC, FCH, F, and CH). As expected, ffPC showed a lower activity compared to PC, FCH and F (**Figure 4B; Table 2**). However, it induced a significantly higher intake compared to STD (*p* < 0.05) and CH (*p* < 0.001), despite the higher calorie content of these two test foods (**Figures 1** and **4B**). Thus, it can be concluded that other determinants trigger the intake of PC in addition to the energy density.

A one-way repeated measures ANOVA was performed to evaluate the influence of the particular test days on the results. Only two experiments showed significant influence of the test days, namely the preference tests PC vs. FCH (*p* = 1.06 × 10^-^^7^) and PC vs. F (*p* = 4.13 × 10^-^^5^) (**Figure 5; Tables 3** and **4**). During the first three test days, the FCH intake by rats, which were naïve to FCH, but had contact with PC in previous tests PC vs. STD, PC vs. F and PC vs. CH, was significantly lower than the PC consumption (*p* < 0.05). On test days 4–6, no significantly higher intake of PC compared to FCH could be observed (*p* > 0.05, **Figure 5A**; **Table 3**). Changes were caused by a clear increase of FCH intake accompanied by a decrease of PC intake over the course of time, whereas the total food intake of both test foods ranged constantly between 70 and 94 g/day during the tests.

**Table 3:** Statistical data of the time dependence of “food intake” for preference tests with the test food combination potato chips (PC) vs. the mixture of fat and carbohydrates (FCH) mean and on test days 1–6.

Food	Mean (%)	SD (%)	df	*p*-Value	*t*-Value	Number of single tests	Number of animals	See figure
Mean
PC	54	12	3	0.5634	0.647	50	18	5A
FCH	46	12
Day 1
PC	89	1	1	0.0118	53.84	6	10	5A
FCH	11	1
Day 2
PC	77	2	1	0.0335	19.00	6	10	5A
FCH	23	2
Day 3
PC	59	0	1	0.0057	111.8	6	10	5A
FCH	41	0
Day 4
PC	57	4	1	0.2593	2.318	6	10	5A
FCH	43	4
Day 5
PC	40	5	1	0.2300	–2.647	6	10	5A
FCH	60	5
Day 6
PC	52	15	1	0.8793	0.192	6	10	5A
FCH	48	15

*Mean and SD of the relative food intake of the independent animal groups (cages) are presented. The *p*-and *t*-values are calculated via two-sided paired Student *t*-test with df as the degree of freedom. Furthermore, number of single tests and number of animals are given.*

**Table 4:** Statistical data of the time dependence of “food intake” for preference tests with the test food combination potato chips (PC) vs. fat (F) mean and on test days 1–6.

Food	Mean (%)	SD (%)	df	*p*-Value	*t*-Value	Number of single tests	Number of animals	See figure
Mean
PC	83	8	3	0.0040	8.050	48	18	5B
F	17	8
Day 1
PC	94	1	1	0.0121	52.68	6	10	5B
F	6	1
Day 2
PC	93	4	1	0.0447	14.23	6	10	5B
F	7	4
Day 3
PC	58	15	1	0.6017	0.723	6	10	5B
F	42	15
Day 4
PC	98	1	1	0.0092	69.00	6	10	5B
F	2	1
Day 5
PC	88	13	1	0.1539	4.057	6	10	5B
F	12	13
Day 6
PC	99	1	1	0.0082	78.00	6	10	5B
F	1	1

*Mean and SD of the relative food intake of the independent animal groups (cages) are presented. The *p*-and *t*-values are calculated via two-sided paired Student *t*-test with df as the degree of freedom. Furthermore, number of single tests and number of animals are given.*

In contrast, no clear trend became apparent when the food intake of PC vs. F was compared on different test days (**Figure 5B; Table 4**).

## DISCUSSION

It was previously shown that snack food such as potato chips is able to modulate brain circuits in rats associated with reward, food intake, satiety, and locomotor activity in comparison to standard chow (Hoch et al., 2013). These modulations of the activity patterns might be responsible for the non-homeostatic intake of snack food.

In studies dealing with non-homeostatic food intake or food addiction, a variety of palatable foods were applied, such as sugar solutions, shortening, cake, potato chips, cookies, or cheese (Prats et al., 1989; Avena et al., 2009; Martire et al., 2013). Usually, food items rich in sugar, fat or both were selected. However, it can be assumed that different types of food and different food components trigger different physiological processes related to food intake. Therefore, it is important to define the exact molecular determinants of a food item that are responsible for the excessive intake and to identify the physiological pathways that are triggered by different food components.

Thus, it was the purpose of the present study to develop a two-choice preference test for screening snack food components for their ability to trigger non-homeostatic food intake. The test system was then applied to investigate how the main macronutrients (carbohydrates and fats) of potato chips contribute to triggering the hedonic intake of this particular snack food.

The induced feeding activity was recorded by two independent readouts. On the one hand, the amount of ingested food or energy (**Figures 3A,B, 4A,B** and **5A,B**; **Tables 1A,B, 2–4**) and, on the other hand, the feeding-related locomotor activity were registered (exemplified in **Figure 3C; Table 1C**). The readout parameters food intake and feeding-related locomotor activity showed a very high correlation (*r* = 0.9204, *R*^2^ = 0.8471, *p* < 0.001). Therefore, it could be excluded that, for example, eventual spillage of the test food biased results.

The absolute amount of consumed food varied from day to day across the different individuals and was also dependent on various further parameters like the age of the animals. Additionally, it had been shown that the reward sensitivity for palatable food is dependent on the development stage of the rats (Friemel et al., 2010). Therefore, a differential two-choice preference test was applied (**Figure 2B**), which recorded the relative food intake of two test foods at a given feeding session. Under these conditions, a training effect could occur due to the presentation of unknown test food versus the known reference food. Therefore, each preference test was performed at least on two different days, i.e., six times. Moreover, the position of the food dispensers containing the test foods was changed after each single test to avoid the development of a place preference. The lack of side- or place-preference was observed by testing STD vs. STD by six consecutive repetitions of a test setting on two consecutive days. Here, no significant difference between the two identical test foods regarding food/energy intake (*p* = 0.3311, **Figures 3A,B; Tables 1A,B**) or feeding related locomotor activity (*p* = 0.5089, **Figure 3C; Table 1C**) was revealed. Finally, in order to minimize the influence of sensory parameters, such as consistency and flavor, the test foods were offered after homogenization in a mixture with powdered STD. Under the applied test conditions, it can, therefore, be concluded that solely differences in the composition of the test foods were responsible for differences in food intake. In summary, the established two-choice preference test seemed to provide reliable results, and could be used to screen for food components related to non-homeostatic food intake.

The developed behavioral test was then applied to investigate the influence of the major components fat and carbohydrates on the potato chip-induced hedonic food intake in ad libitum fed rats. The first experiment confirmed that PC induced a higher food and energy intake than STD indeed (**Figures 3A,B; Tables 1A,B**). As expected, a higher food intake compared to STD was also observed when the isolated potato chip components fat and carbohydrates were offered in similar concentrations as present in potato chips (**Figure 4A; Table 2**). It is worth noting that the fat component was more active than the carbohydrate component. Consequently, it can be concluded that fat seems to be one contributor to the palatability of a test food. It is reported that the preference of rats for fat is learned and leads to a preference for fatty food: rats fed a high-fat diet showed an enhanced intake of oil emulsions compared to rats which received a diet high in carbohydrates (Reed and Friedman, 1990). Beside this influence on food preference, fat is a strong contributor to an enhanced food intake by additionally increasing the meal size (Warwick and Synowski, 1999).

However, the effects of fat intake seem to be rather complex. Fat (corn oil) in the oral cavity of mice likely led to activation of the dopaminergic system through dopamine D1 receptor, which seemed to be a mediator of its reinforcing effects (Imaizumi et al., 2000). Possibly, the fatty acid transporter CD36 is involved in the detection of dietary fats in the oral cavity of rats or mice. This early detection of fats might lead to a quick preference for fatty foods (Laugerette et al., 2005).

Additionally, post-ingestive effects are responsible for an increased intake of fat. It was shown in a self-regulated intragastric infusion paradigm that rats take up a higher amount of a high-fat diet compared to a high-carbohydrate diet via intragastric infusion (Warwick et al., 2003). Such post-ingestive effects of fats are possibly mediated by fatty acid sensors such as CD36, GPR40, and GPR120 in the small intestine leading to a post-oral stimulation of appetite (Sclafani and Ackroff, 2012; Sclafani et al., 2013).

However, in the present study, neither the fat component, nor the carbohydrate component alone was able to induce food intake similar to PC. Only the combination of both components (FCH) led to a food/energy intake comparable to PC suggesting a synergistic effect of fats and carbohydrates (**Figures 3A,B; Tables 1A,B**). Consequently, FCH induces higher food intake than F, CH, or STD (**Figure 4A; Table 2**). A previous study with two different groups of rats showed that the group which had access to a mixed food consisting of fat and carbohydrates ingested a larger quantity of food compared to a group of rats which were provided with food solely with high fat content (Ramirez and Friedman, 1990). This result is in accordance with the present outcome of our two-choice preference test on solid snack food. Preference tests with liquid test food already showed that rats preferred an emulsion with fat and sugar over the single components as well as over standard chow (Lucas and Sclafani, 1990).

From these findings, it can be hypothesized that the combination of the macronutrients, fat and carbohydrates, triggers additional effects compared to the administration of only one of the components. One study showed, for example, that in rats, the administration of the GABA-B receptor agonist baclofen stimulated binge-eating of sweet-fat food, suppressed binge-eating of fat, but had no effect on binge-eating of sucrose (Berner et al., 2009). These findings clearly indicate the presence of specific mechanisms related to excessive intake of different macronutrients or their combination. Moreover, a study with rats by la Fleur et al. (2010) observed that a mixture of fat and sugar, but not the single components, led to hyperphagia-induced obesity. Additionally, the mixture of fat and sugar altered hypothalamic neuropeptide expression in a different way compared to fat or sugar alone (la Fleur et al., 2010).

Since the test foods were tested against each other in different combinations, situations could occur that animals were familiar with test foods from previous preference tests, but naïve to a newly introduced test food. Thus, the novelty or the familiarity of a test food could influence the food intake. Therefore, preference tests were performed at least six times, so that the animals were familiar with both test foods already after the first test. Subsequent ANOVA analysis revealed that the variable “test day” did not have a significant influence except for the preference tests PC vs. FCH and PC vs. F. Interestingly, a clear trend was observed in the PC vs. FCH combination: the rats, which were familiar with PC from previous preference tests during this study (PC vs. STD, F or CH), significantly preferred PC over FCH in the first three test days (*p* < 0.05). In the following test days, the preference for PC diminished (**Figure 5A; Table 3**). Thus, it can be concluded that FCH and PC have similar capability to induce food intake in ad libitum fed rats, but PC were preferred when rats were naïve to FCH but not to PC. In contrast, no clear trend was observed when PC was tested against F. Instead, a high and constant preference of PC against F was observed on five out of six test days. Therefore, the novelty of a particular test food did not seem to influence the feeding preference in general, but only when PC was tested against FCH.

Additionally to novelty effects, the order of food presentation could influence the feeding behavior. For example, food fatigue or acclimation could occur. Therefore, some preference tests, which had been performed at the beginning of the study, were repeated at the end of the whole sequence (e.g., PC vs. F, PC vs. CH). The repetitions provided results very similar to the initial tests. However, it cannot be fully excluded that food fatigue or acclimation effects occur under the applied conditions.

The capability of the test foods STD, CH, F, and FCH to induce food intake may be an effect of their respective energy density, because the test foods that induced higher food intake often had higher calorie content (**Figure 1**). However, the experiments with ffPC indicate that the energy content is apparently not the only trigger of food intake in non-deprived animals. The presentation of ffPC led to a significantly lower additional food intake compared to regular PC (*p* < 0.001, **Figure 4B; Table 2**). These results suggest that appetent fat intake is less related to textural fat properties, such as mouth feeling, but rather to the caloric content or the chemoreception of free fatty acids in the digestive tract or the gustatory system (Pittmann, 2010). In contrast to this finding, it has been reported previously that no preference could be observed in non-deprived rats for high-fat cake compared to no-fat cake. Only food-deprived rats highly preferred the high-fat cake (Sclafani et al., 1993). Notably, ffPC were highly preferred over STD and CH despite of the lower energy density of ffPC (**Figure 4B; Table 2**). Hence, other components or properties of ffPC beyond the energy content seem to have an additional influence on the activity of snack food to induce food intake. For example, salt or fiber may affect the food intake (Beauchamp and Bertino, 1985; Vitaglione et al., 2009). The two-choice preference test that has been applied in the present study may now provide a useful screening system to further investigate the (minor) components of potato chips which contribute to their non-homeostatic intake. The conclusion that the energy content is not the only parameter inducing food intake is supported by a previous study in which the addition of saccharin to a fat emulsion had a similar enhancing effect on food intake as the addition of sucrose (Lucas and Sclafani, 1990).

In conclusion, the present study established a behavioral screening tool that has been optimized to investigate the ability of different test foods to induce food intake in ad libitum fed rats. The assay was used to examine how the main macronutrients of potato chips, namely fat and carbohydrates, contribute to trigger hedonic food intake. It was shown that fat has a high impact on additional food intake, but the combination of both macronutrients was identified as the main contributor to the palatability of potato chips. The energy density is not the sole factor responsible for the increased food intake, since ffPC triggered higher food intake than other test foods with higher energy content. The two-choice preference test used in this study will be applied in future investigations to disentangle the influence of minor components of potato chips so that the molecular determinants of their intake can be understood in more detail. Additionally, it should be investigated if a mixture of fat and carbohydrates is able to induce similar changes in brain activity patterns as snack food.

## AUTHOR CONTRIBUTIONS

Conceived and designed the experiments: Tobias Hoch, Monika Pischetsrieder, Andreas Hess. Performed the experiments and analyzed the data: Tobias Hoch. Interpreted the data: Tobias Hoch, Monika Pischetsrieder, Andreas Hess. Contributed reagents/materials/analysis tools: Monika Pischetsrieder, Andreas Hess. Wrote the paper: Tobias Hoch, Monika Pischetsrieder, Andreas Hess. Finally approved of the version to be published: Tobias Hoch, Monika Pischetsrieder, Andreas Hess. Agreed to be accountable for all aspects of the work in ensuring that questions related to the accuracy or integrity of any part of the work are appropriately investigated and resolved: Tobias Hoch, Monika Pischetsrieder, Andreas Hess.

## Conflict of Interest Statement

The authors declare that the research was conducted in the absence of any commercial or financial relationships that could be construed as a potential conflict of interest.
